# Prevalence and associated factors of denture use among older adults living in rural and urban areas of China: a national cross-sectional study

**DOI:** 10.1186/s12903-025-05684-1

**Published:** 2025-03-21

**Authors:** Yuxin Eva Lin, Xiaoyue Zhang, Meijun Chen, Ying Ji, Yuhui Shi, Yuting Lin, Xiaochen Yang, Wangnan Cao, Chun Chang

**Affiliations:** 1 School of Public Health, Peking University, Beijing, 100191 China; 2 School of Public Health, the University of Hong Kong, Hong Kong, 999077 China

**Keywords:** Older people, Oral health, Tooth loss, Denture, Urban-rural gap

## Abstract

**Backgrounds:**

Tooth loss, often considered as an inevitable result of ageing, is one of the most frequently stated problems with older adults worldwide, which entails a negative impact on physical and mental health, as well as quality of life. However, there is urban-rural gap among older adults in both oral health condition and dental service utilization. The study focused on the urban-rural gap in Chinese older population, aiming to describe their tooth loss and denture use status, and explore the associated factors, hoping to provide insights into improving the oral healthcare system in China and other developing countries.

**Methods:**

This cross-sectional study used data collected from May to September in 2020. A stratified, multi-stage cluster sampling design was used to produce nationally representative samples of 2709 adults aged 60 years and older. The dependent variable was denture use of the participant. Determinants included demographic characteristics, health status, social support, oral health knowledge and health service utilization. Descriptive analysis was conducted to describe the sample characteristics, and Generalized Linear Mixed Model was used to identify independent factors associated with denture use among older adults in rural and urban China respectively.

**Results:**

There were significant urban-rural differences in tooth loss, denture use and health service utilization among Chinese older adults. Urban participants’ denture use was associated with oral health knowledge (OR = 1.29, 95%CI = 1.07–1.56). Rural participants’ denture use was associated with oral health knowledge (OR = 1.23, 95%CI = 1.02–1.47), dental checkup in the last six months (OR = 1.19, 95%CI = 1.00-1.40), and social support (OR = 1.22, 95%CI = 1.03–1.44).

**Conclusions:**

Greater efforts should be made to enhance oral health education and emphasize the importance of regular dental checkups among older population. Targeted focus on different aspects of oral health based on the characteristics of different populations is suggested. Policymakers should be aware of the urban-rural disparity in distribution and utilization of oral health services.

## Background

Oral health inequality, according to the World Health Organization, is an issue based on professional practice, ethical public health policy, and social justice [1]. It is a burden of oral disease, which disproportionately affecting people from more disadvantaged backgrounds and vulnerable populations, like older adults. As the United Nations declared in *Decade of Healthy Ageing (2021–2030)*, oral health is a key indicator of overall health in older age [2]. For older adults, poor oral health is linked with non-communicable diseases (NCDs), overall frailty and reduced quality of life [3, 4]. Previous study has shown the urban-rural gap among older adults in both oral health condition and dental service utilization [5]. Therefore, it is crucial to address oral health inequality in the older population.

Tooth loss, often considered as an indicator in the irreversible process of ageing, is one of the most frequently stated problems with older adults worldwide [6]. For people over 60 years old, almost one in four persons suffer from complete toothlessness or edentulism [1]. As the end point of a lifelong history of oral disease, it can limit chewing ability and nutrient intake, affect appearance and articulation, which entails a negative impact on physical and mental health, as well as social function and quality of life [7–10]. In the global concerns, the burden of disability-adjusted life years (DALYs) due to tooth loss is estimated to be over 9.5 million [1].

Dentures, as a widely accepted treatment for tooth loss, are prostheses that can replace one or more natural teeth [11]. A number of studies have shown that the harm of tooth loss can be partially counteracted by using dentures, especially in chewing and swallowing function, cognitive health, life expectancy, and quality of life [7, 11–13]. According to the 7th National Population Census in 2020, people aged 60 and older in China were 264 million, accounting for 18.7% of the total population [14]. Among Chinese older population aged 65–74, the average number of remaining teeth was 22.5 in 2015 [15]. However, with none of the basic medical insurance plans covering the cost of dentures, only 63.2% of Chinese older adults with missing teeth use dentures [15]. Additionally, while much is known about oral health service utilization, less attention has been paid to the factors influencing denture use among older adults in China.

In 2018, the National Investigation of Resources for Oral Health in China has showed insufficiencies and inequalities in distribution of stomatological staff and institutions nationally. Also, surveys conducted by the National Health Commission (2015) have established the significant distinction between rural and urban Chinese seniors, in terms of the proportion of tooth loss and denture use [15]. Prior studies have also suggested that the frequency of dental checkups and the expenditure on dental care among older adults varies greatly between rural and urban areas, alongside the difference in its relevant factors [16–18]. However, a gap remains in understanding the specific factors contributing to these differences, both in China and abroad. Addressing on this topic is critical for promoting urban-rural health equality, which can also serve as a reference for other developing countries.

Therefore, in this study, we focused on the urban-rural gap in Chinese older population, aiming to describe their tooth loss and denture use status, and explore the associated factors. By highlighting the disparities between rural and urban China, we hope to provide insights into improving the oral healthcare system in China and other developing countries.

## Methods

### Study design

This cross-sectional study used data collected from May to September in 2020. A stratified, multi-stage cluster sampling design was used to produce nationally representative samples of adults aged 60 years and older. First, we selected six provinces/municipalities (Zhejiang, Jiangxi, Beijing, Gansu, Chongqing, and Liaoning) out of the Six Administrative Regions of mainland China (East, South Central, North, Northwest, Southwest, and Northeast, correspondingly). Second, from each province/municipality, we selected the provincial capital city and randomly selected another city whose Gross Domestic Product (GDP) last year was in the bottom third of the province/municipality. Then, we randomly selected a district (urban) and a county (rural) respectively from each city above. From each district/county, a sub-district/town was also selected randomly. Finally, we conducted the survey in the selected sub-districts and towns, the proportion of the population surveyed was determined according to the urbanization rate of each city.

### Population

The participants in this study were recruited from community health centers in collaboration with the family doctors. The inclusion criteria were as follows: (1) 60 years old or above; (2) local residents who had been living in the selected area for more than 6 months prior to the start of the study; (3) signed the informed consent form. Participants who had mental disorders or dementia were excluded from the study.

No less than 250 questionnaires were collected from each city selected; overall, a total of 3056 participants completed the questionnaires. 60 (1.96%) questionnaires were excluded for illegible handwriting. Our analyses used all 2709 participants for whom the variables of interest were available, with no imputation for missing data.

The study was conducted according to the guidelines of the Declaration of Helsinki and approved by the Peking University Health Science Center (IRB00001052-19143). All participants provided written or oral informed consent.

### Data collection

In principle, the questionnaires should be completed by participants themselves according to their own understanding; if the participant had difficulties in reading or writing, and could not complete the questionnaire independently, an investigator would ask the questions in person. Inductive or suggestive language was forbidden. Informed consents were obtained before participation.

The questionnaire and work procedure were designed by the research team in Peking University. All investigators received standard training before the survey. Investigators were required to check every questionnaire item on the spot to avoid any error and ensure data quality; quality control personnel were required to review all the questionnaires on a daily basis.

### Variables

#### Dependent variable

Denture use of participants was measured by the question “Do you have any missing teeth?” and were required to choose one among four options described below: “No, I don’t have any missing teeth”, “Yes, and I am currently using dentures”, “Yes, and I have intention to use dentures”, and “Yes, and I don’t have intention to use dentures”. In this question, “denture” refers to any types of dental prostheses, including implanted, fixed and removable ones. We excluded those participants who had no missing teeth in subsequent analysis; then, we defined the second option as “using dentures”, and the last two options were named “without using dentures”. Thus, the participants were divided into two groups by this question.

#### Independent variables

Independent variables consisted of demographic characteristics, oral health knowledge, dental checkups, community healthcare and health education service utilization, social support, and health related quality of life.

##### Demographic characteristics

Demographic characteristics included participants’ age, gender, education level, marital status, household income, and urban/rural registered residence.

##### Dental checkups in 6 months

Dental checkups were measured by whether the participant took dental checkups (including preventive and therapeutic checkups) in the 6 months prior to data collection.

##### Oral health knowledge

In participants’ knowledge of basic oral health for older adults, we used a self-designed multiple-choice question, which was developed based on *the Bulletin of the General Office of the National Health and Family Planning Commission on the Issuance of Core Information on the Health of Older Adults* issued in 2014 [19]. In this question, we simultaneously tested participants’ knowledge of dental cavity repairment, missing teeth replacement, and the frequency of dental checkups recommended by experts. The scores were separated into a low and a high level group by median split.

##### Community healthcare service utilization and community health education service utilization

To assess community healthcare and health education service utilization, we used a set of questions to gauge how often the participants use community healthcare services (free medical consultations, family doctors, physical examinations, exp.) and community health education services (health lectures, health message billboards, health promotion materials, and in-person health education, exp.). Each question used a 4-point Likert scale with 1 = never and 4 = always. Items were scored and summed, then separated into a low and a high level group by median split.

##### Social support

Social support level was measured by the Social Support Rating Scale (SSRS), which has been applied in a wide range of Chinese populations because of its good reliability and validity, with a Cronbach’s α of 0.69 [20]. The Scale consisted of 10 items, including objective support (3 items), subjective support (4 items) and the utilization of social support (3 items), with 3 scores in these 3 dimensions, as well as a total social support score ranging from 12 to 66. The scores were also separated into a low and a high level group by median split.

##### HRQoL and self-assessed health status

In order to measure participants’ health related quality of life (HRQoL), the EuroQoL-5 Dimensions (EQ-5D) and Visual Analogue Scale (VAS) system were applied. EQ-5D consists of 5 dimensions (mobility, self-care ability, daily activities, pain/discomfort, and anxiety/depression), with each dimension having three possible outcomes (no problems, moderate problems and extreme problems) [21, 22]. The answers were grouped by “having no problem in any of the 5 dimensions” and “having some problems in any of the 5 dimensions”. The EQ-VAS asked the participant to consider their health state during the past one month, then provides an assessment of current health status on a vertical scale of 0–100, with a higher score indicated better self-assessed health status [21, 22]. The scores were divided into tertiles: lower, medium and higher self-assessed health status.

### Data analysis

The descriptive analysis was used to describe the sample characteristics based on rural and urban registered residence. A chi-square test was applied to explore whether the distribution of variables was significantly different between these two subgroups. By taking the sampling cluster into consideration, Generalized Linear Mixed Model (with package lme4) was conducted to identify independent factors associated with denture use among older adults in rural and urban China respectively. All *P* values reported are two-tailed. Statistical significance was set at 0.05. Data analyses were performed using R 4.4.1.

## Results

### Sample characteristics

A total of 2709 participants were included in the analysis Tables 1 and 2: 52.01% (1,409/2,709) were urban residents, and 47.99% (1,300/2,709) were rural residents. The overall prevalence of tooth loss was 77.2% (2092), in which 890 participants never replaced their missing teeth, accounting for 32.85% of the total sample population.

There were some significant urban-rural differences in most of the assessed variables: For rural participants, 80.54% (1047) had missing teeth, 38.62% (502) didn’t use dentures, with proportions of 74.17% (1045) and 27.54% (388), correspondingly, in urban participants. Compared with urban participants, rural participants were significantly older, more likely to be male, less educated, widowed, from less affluent families, and self-reported less healthy; Besides, rural participants had relatively lower levels of oral health knowledge, Community healthcare service utilization, social support (and its dimension utilization of social support), and dental checkups than urban participants (all *P* <.05). For health-related quality of life, there was no significant difference between rural versus urban participants. After stratified by age groups, in 2 out of 4 age groups, the rate of missing teeth is significantly higher in rural participants than urban ones (65–69: 68.29% vs. 76.43%, *P* =.008; 70–74: 76.72% vs. 83.16%, *P* =.031).

**Table 1:** Sociodemographic characteristics of the study population, urban versus rural registered residence

Variables	Overall(*N* = 2709) *n*(%)		Urban(*N* = 1409) *n*(%)		Rural(*N* = 1300) *n*(%)		chi-square	*P* value^b^
Province/municipality								
Zhejiang	475	(17.53)	329	(23.35)	146	(11.23)	108.190	<0.001^***^
Jiangxi	458	(16.91)	200	(14.19)	258	(19.85)		
Beijing	473	(17.46)	248	(17.60)	225	(17.31)		
Gansu	469	(17.31)	276	(19.59)	193	(14.85)		
Chongqing	408	(15.06)	181	(12.85)	227	(17.46)		
Liaoning	426	(15.73)	175	(12.42)	251	(19.31)		
Age group							9.692	0.021^*^
60–64	391	(14.43)	226	(16.04)	165	(12.69)		
65–69	854	(31.52)	451	(32.01)	403	(31.00)		
70–74	722	(26.65)	348	(24.70)	374	(28.77)		
75+	742	(27.39)	384	(27.25)	358	(27.54)		
Gender							10.941	<0.001^***^
Male	1205	(44.48)	584	(41.45)	621	(47.77)		
Female	1504	(55.52)	825	(58.55)	679	(52.23)		
Education level							628.683	<0.001^***^
Primary school or less	1332	(49.17)	383	(27.18)	949	(73.00)		
Middle school	792	(29.24)	529	(37.54)	263	(20.23)		
High school	410	(15.13)	326	(23.14)	84	(6.46)		
College or higher	175	(6.46)	171	(12.14)	4	(0.31)		
Marital status							21.385	<0.001^***^
Never married	12	(0.44)	6	(0.43)	6	(0.46)		
Married	2215	(81.76)	1162	(82.47)	1053	(81.00)		
Divorced	53	(1.96)	42	(2.98)	11	(0.85)		
Widowed	429	(15.84)	199	(14.12)	230	(17.69)		
Household income^a^							951.926	<0.001^***^
Low	700	(25.84)	40	(2.84)	660	(50.77)		
Low and middle	1296	(47.84)	756	(53.66)	540	(41.54)		
Upper middle	596	(22.00)	516	(36.62)	80	(6.15)		
High	117	(4.32)	97	(6.88)	20	(1.54)		
Basic medical insurance							1612.757	<0.001^***^
Urban Employee Basic Medical Insurance	909	(33.55)	887	(62.95)	22	(1.69)		
Urban Resident Basic Medical Insurance	741	(27.35)	380	(26.97)	361	(27.77)		
Government medical insurance	80	(2.95)	73	(5.18)	7	(0.54)		
New Cooperative Medical Scheme	954	(35.22)	58	(4.12)	896	(68.92)		
None of the above	25	(0.92)	11	(0.78)	14	(1.08)		

a. Income level was classified as low, low and middle, upper middle, and high. For urban residents (personal monthly income): low, RMB < 600 (USD < 91); low and middle, RMB 600 ∼ 3500 (USD 91 ∼ 533); upper middle, RMB 3500 ∼ 6500 (USD 533 ∼ 990), and high, RMB ≥ 6500 (USD ≥ 990). For rural residents (household annually income): low, RMB < 17,000 (USD 2589); low and middle, RMB 17,000 ∼ 65,000 (USD 2589 ∼ 9900); upper middle, RMB 65,000 ∼ 100,000 (USD 9900 ∼ 15,231), and high, RMB ≥ 100,000 (USD ≥ 15,231). The same hereinafter

b. *P <.05; **P <.01; ***P <.001. The same hereinafter

**Table 2:** Health-related characteristics of the study population, urban versus rural registered residence

Variables	Overall(*N* = 2709) *n*(%)		Urban(*N* = 1409) *n*(%)		Rural(*N* = 1300) *n*(%)		*p* value
Dental checkup in 6 months							<0.001^***^
Yes	584	(21.56)	411	(29.17)	173	(13.31)	
No	2125	(78.44)	998	(70.83)	1127	(86.69)	
Oral health knowledge							<0.001^***^
Correct	1490	(55.00)	952	(67.57)	538	(41.38)	
Wrong	1219	(45.00)	457	(32.43)	762	(58.62)	
Community health education service utilization							<0.001^***^
Low level	960	(35.44)	443	(31.44)	517	(39.77)	
High level	1749	(64.56)	966	(68.56)	783	(60.23)	
Community healthcare service utilization							0.005^**^
Low level	818	(30.20)	392	(27.82)	426	(32.77)	
High level	1891	(69.80)	1017	(72.18)	874	(67.23)	
Social support							
Objective support							0.920
Low level	1506	(55.59)	782	(55.50)	724	(55.69)	
High level	1203	(44.41)	627	(44.50)	576	(44.31)	
Subjective support							0.169
Low level	1528	(56.40)	777	(55.15)	751	(57.77)	
High level	1181	(43.60)	632	(44.85)	549	(42.23)	
Utilization of social support							<0.001^***^
Low level	1530	(56.48)	746	(52.95)	784	(60.31)	
High level	1179	(43.52)	663	(47.05)	516	(39.69)	
Total score							0.045^*^
Low level	1244	(45.92)	621	(44.07)	623	(47.92)	
High level	1465	(54.08)	788	(55.93)	677	(52.08)	
Self-assessed health status							<0.001^***^
Lower	858	(31.67)	394	(27.96)	464	(35.69)	
Medium	908	(33.52)	472	(33.50)	436	(33.54)	
Higher	943	(34.81)	543	(38.54)	400	(30.77)	
HRQoL							0.247
Have no problem	1805	(66.63)	953	(67.64)	852	(65.54)	
Have some problems	904	(33.37)	456	(32.36)	448	(34.46)	
Missing teeth and denture use							<0.001^***^
Have no missing teeth	617	(22.78)	364	(25.83)	253	(19.46)	
Have used dentures	1202	(44.37)	657	(46.63)	545	(41.92)	
Intent to use dentures	333	(12.29)	172	(12.21)	161	(12.38)	
Have no intention to use dentures	557	(20.56)	216	(15.33)	341	(26.23)	

### Characteristics associated with denture use

Urban participants’ denture use was associated with oral health knowledge (OR = 1.29, 95%CI = 1.07–1.56). Rural participants’ denture use was associated with oral health knowledge (OR = 1.23, 95%CI = 1.02–1.47), dental checkup in the last six months (OR = 1.19, 95%CI = 1.00-1.40), and social support (OR = 1.22, 95%CI = 1.03–1.44). Results are presented in Table 3.

**Table 3:** Factors associated with denture use among urban versus rural registered residence

Variables	Urban			Rural		
	OR	95% CI	*P* value	OR	95% CI	*P* value
(Intercept)	0.31	0.15–0.66	0.002^**^	0.36	0.19–0.67	0.002^**^
Age group						
60–64	ref			ref		
65–69	1.03	0.75–1.41	0.854	1.11	0.86–1.42	0.433
70–74	1.21	0.88–1.65	0.236	1.15	0.89–1.49	0.285
75+	1.23	0.89–1.69	0.205	1.10	0.85–1.43	0.472
Gender						
Male	ref			ref		
Female	1.06	0.89–1.27	0.508	1.04	0.88–1.23	0.639
Education level						
Primary school or less	ref			ref		
Middle school	0.98	0.78–1.22	0.833	1.01	0.81–1.25	0.951
High school	0.99	0.69–1.41	0.956	1.10	0.87–1.40	0.424
College or higher	0.85	0.20–3.61	0.825	0.98	0.73–1.32	0.919
Marital status						
Married	ref			ref		
Others	0.97	0.77–1.22	0.781	1.08	0.87–1.34	0.471
Household income						
Low	ref			ref		
Low and middle	1.04	0.86–1.26	0.673	1.12	0.67–1.89	0.664
Upper middle	1.06	0.74–1.51	0.743	1.08	0.63–1.85	0.779
High	0.73	0.27-2.00	0.547	1.17	0.64–2.12	0.613
Basic medical insurance						
None of the following	ref			ref		
Urban Employee Basic Medical Insurance	1.30	0.69–2.43	0.412	0.87	0.72–1.06	0.163
Urban Resident Basic Medical Insurance	0.95	0.26–3.52	0.939	0.86	0.60–1.22	0.397
Government medical insurance	1.03	0.55–1.92	0.919	0.76	0.47–1.23	0.258
New Cooperative Medical Scheme	1.04	0.35–3.07	0.947	1.00	0.44–2.29	0.992
Oral health knowledge						
Low level	ref			ref		
High level	**1.29**	**1.07–1.56**	0.009^**^	**1.23**	**1.02–1.47**	0.028^*^
Dental checkup in 6 months						
No	ref			ref		
Yes	1.17	0.94–1.47	0.157	**1.19**	**1.00-1.40**	0.044^*^
Community health education service utilization						
Low level	ref			ref		
High level	0.99	0.81–1.20	0.890	1.07	0.89–1.29	0.489
Community healthcare service utilization						
Low level	ref			ref		
High level	1.00	0.82–1.21	0.964	1.01	0.84–1.22	0.886
Social support						
Low level	ref			ref		
High level	1.11	0.92–1.34	0.281	**1.22**	**1.03–1.44**	0.018^*^
Self-assessed health status						
Lower	ref			ref		
Medium	0.96	0.78–1.19	0.713	1.08	0.88–1.32	0.467
Higher	1.14	0.92–1.43	0.234	1.01	0.82–1.25	0.903
HRQoL						
Have no problem	ref			ref		
Have some problems	1.03	0.85–1.25	0.743	0.92	0.77–1.09	0.331

## Discussion

Our study is the first to focus on the urban-rural gap of tooth loss and denture use among Chinese older adults, describe the current status of missing teeth and denture use, and explore the associated factors. According to our results, there are significant differences in the status of missing teeth and denture use between rural and urban older Chinese population; Oral health knowledge was found to be associated with denture use in both rural and urban older adults. Additionally, our findings indicate the difference between factors associated with the denture use of urban versus rural older adults: for rural older adults, denture use is also related to dental checkups and social support levels; meanwhile, for urban ones, no other explanatory variables were found to be statistically significant.

The findings of this study showed significantly lower levels in oral health knowledge, dental checkups, denture use, community healthcare and health education service utilization among the rural elderly population; Plus, there was a significant gap between rural and urban older adults in tooth loss status, even if the influence of age was controlled. These results corroborate the findings of a great deal of the previous work in oral health status and oral health care, thus confirm the idea that urban-rural disparities are common in the field of oral health [16–18, 23]. Oral health promotion in rural areas is more challenging than in urban areas.

Consistent with previous studies of oral health care utilization, the current study found that oral health knowledge levels were associated with denture use among both urban and rural older adults, and dental checkups were also important factors for rural ones [24–26]. This reflects the importance of health education and regular dental checkups. In fact, the “Oral Health Guide for Chinese Residents: Older Adults” released by the National Health Commission in 2009 emphasizes that older adults should “have oral health checkups at least once every six months” and “have a relatively complete dentition” [27]. In spite of that, this survey still revealed a lack of oral health knowledge and behaviors among older adults. A part of this could be due to the insufficiency and uneven distribution of dental clinics nationally [28].

In addition to the difference in the current status of denture use, it’s interesting to note that there are also additional factors associated with denture use among rural older adults, while we failed to find one for urban ones. For rural older adults, higher level of social support was associated with more denture use. In accordance with the present result, previous studies have demonstrated that social interaction and participation may provide older adults with motivation and resources to promote oral health care utilization [29–32]. People with poor oral health are more likely to be socially isolated, which is another possible explanation. This may suggest that rural older adults are motivated to use dentures more by their social life. As a consequence, it might be efficient for health educators to provide additional types of health-promoting services in rural areas: For example, engaging rural older adults in oral health activities with much interaction, and organizing free dental checkups in rural areas.

In contrast to earlier findings, however, the results of this study did not show any statistically significant association in other personal characteristics such as the household income. This discrepancy could be attributed to the difference between denture use and other oral health care utilization, for example, the price of different types of dentures varies widely, and there are products available regardless of income. Additionally, the findings of the current study are contrary to previous studies which have suggested that better dental insurance plans promote the older adults’ oral health care utilization [18, 33–35]. This inconsistency may be due to the fact that denture fitting is still considered as a cosmetic medical service in the current medical system, and none of the basic medical insurance includes dentures in their coverage.

There were some limitations in the current study that have to be considered, however. First, given its cross-sectional study design, no causal relationships can be established between denture use and underlying determinants. Second, need factors such as number of missing teeth and effect of tooth loss on oral function were not available from the survey, consequently these factors were not included in the analyses. Research shows that chewing ability of older adults is affected not only by the number of remaining teeth, but also their location and distribution [36]; The retention of not less than 20 fully-functional natural teeth is sufficient to meet most oral functional demands, such as oral chewing function, aesthetics, articulation and social interaction [36, 37]. Thus, the need for dentures should be individualized. Third, older adults with mental disorders or dementia, whose health status strongly affected by denture use, were excluded from the study. Older adults with cognitive impairment are more likely to be edentulous, however, when they lose their teeth, they are also more unlikely to use dentures, even if research has confirmed that denture use is able to counteract the negative impact of tooth loss on cognitive function [12, 38]. Finally, since the data collected was self-reported, recall bias might have been introduced.

Notwithstanding these limitations, this study offers valuable insights into factors relevant to denture use and urban-rural disparities. Further studies could pay attention to the differences in various types of non-natural teeth, as partial or complete, removable or implant-retained fixed dentures. Moreover, the issue of denture use among older adults with poor self-care ability is also an intriguing one which could be usefully explored in further research.

Therefore, greater efforts should be made to enhance oral health education and emphasize the importance of regular dental checkups among older population. Targeted focus on different aspects of oral health based on the characteristics of different populations is suggested. Another important practical suggestion is that the policymakers should be aware of the urban-rural disparity in distribution and utilization of oral health services. A reasonable approach to tackle this issue could be to accelerate the construction of dental healthcare institution in rural areas, and expand basic medical insurance coverage for necessary denture fitting services.

## Conclusion

There are significant differences in the status of missing teeth and denture use between rural and urban older Chinese population; Oral health knowledge and dental checkups were found to be associated with denture use in both rural and urban older adults. Urban older adults’ denture use correlates with social support levels, while rural older adults’ denture use is associated with their age and self-assessed health. Greater efforts should be made to enhance oral health education and emphasize the importance of regular dental checkups among older population, as well as explore the urban-rural disparity in distribution and utilization of oral health services.

## Data Availability

The datasets generated and analyzed during the current study are not publicly available due to participants’ privacy reasons but are available from the corresponding author on reasonable request.

## Abbreviations

NCDs: Non-Communicable Diseases
DALYs: Disability-Adjusted Life Years
GDP: Gross Domestic Product
HRQoL: Health Related Quality of Life
EQ-5D: The EuroQoL-5 Dimensions
VAS: Visual Analogue Scale
